# Supplementary material for: Resolving the full spectrum of human genome variation using Linked-Reads
Source: Genome Res. 2019 Apr;29(4):635–45. doi: 10.1101/gr.234443.118 (PMC6442396; doi:10.1101/gr.234443.118)
Supplement: Supplemental Material [file supp_gr.234443.118_Supplemental_longranger-2.2.zip › longranger-2.2.tar.bz2!/lr2.2-public/lib/stan/cmdstan/stan/doc/api/html/namespacemembers_g.html]

Stan: Namespace Members


|  |  |
| --- | --- |
|  | Stan  2.5.0  probability, sampling & optimization |


- Main Page
- Related Pages
- Namespaces
- Classes
- Files

- Namespace List
- Namespace Members

- All
- Functions
- Variables
- Typedefs
- Enumerations
- Enumerator

- a
- b
- c
- d
- e
- f
- g
- h
- i
- j
- l
- m
- n
- o
- p
- q
- r
- s
- t
- u
- v
- w


All Classes Namespaces Files Functions Variables Typedefs Enumerations Enumerator Friends Macros Pages

Here is a list of all namespace members with links to the namespace documentation for each member:

### - g -

- gamma\_ccdf\_log()
  : stan::prob
- gamma\_cdf()
  : stan::prob
- gamma\_cdf\_log()
  : stan::prob
- gamma\_log()
  : stan::prob
- gamma\_p()
  : stan::agrad
  , stan::math
- gamma\_q()
  : stan::agrad
  , stan::math
- gamma\_rng()
  : stan::prob
- gaussian\_dlm\_obs\_log()
  : stan::prob
- generate\_arg\_decl()
  : stan::gm
- generate\_bare\_type()
  : stan::gm
- generate\_class\_decl()
  : stan::gm
- generate\_comment()
  : stan::gm
- generate\_constrained\_param\_names\_method()
  : stan::gm
- generate\_constructor()
  : stan::gm
- generate\_cpp()
  : stan::gm
- generate\_destructor()
  : stan::gm
- generate\_dims\_method()
  : stan::gm
- generate\_end\_class\_decl()
  : stan::gm
- generate\_end\_namespace()
  : stan::gm
- generate\_expression()
  : stan::gm
- generate\_function()
  : stan::gm
- generate\_function\_arguments()
  : stan::gm
- generate\_function\_body()
  : stan::gm
- generate\_function\_functor()
  : stan::gm
- generate\_function\_inline\_return\_type()
  : stan::gm
- generate\_function\_name()
  : stan::gm
- generate\_function\_template\_parameters()
  : stan::gm
- generate\_functions()
  : stan::gm
- generate\_functor\_arguments()
  : stan::gm
- generate\_include()
  : stan::gm
- generate\_includes()
  : stan::gm
- generate\_indent()
  : stan::gm
- generate\_indexed\_expr()
  : stan::gm
- generate\_init\_method()
  : stan::gm
- generate\_init\_vars()
  : stan::gm
- generate\_initialization()
  : stan::gm
- generate\_initializer()
  : stan::gm
- generate\_local\_var\_decls()
  : stan::gm
- generate\_local\_var\_init\_nan()
  : stan::gm
- generate\_local\_var\_inits()
  : stan::gm
- generate\_log\_prob()
  : stan::gm
- generate\_member\_var\_decls()
  : stan::gm
- generate\_member\_var\_inits()
  : stan::gm
- generate\_model\_name\_method()
  : stan::gm
- generate\_model\_typedef()
  : stan::gm
- generate\_param\_names\_method()
  : stan::gm
- generate\_printable()
  : stan::gm
- generate\_private\_decl()
  : stan::gm
- generate\_propto\_default\_function()
  : stan::gm
- generate\_propto\_default\_function\_body()
  : stan::gm
- generate\_public\_decl()
  : stan::gm
- generate\_set\_param\_ranges()
  : stan::gm
- generate\_start\_namespace()
  : stan::gm
- generate\_statement()
  : stan::gm
- generate\_statements()
  : stan::gm
- generate\_type()
  : stan::gm
- generate\_typedef()
  : stan::gm
- generate\_typedefs()
  : stan::gm
- generate\_unconstrained\_param\_names\_method()
  : stan::gm
- generate\_using()
  : stan::gm
- generate\_using\_namespace()
  : stan::gm
- generate\_usings()
  : stan::gm
- generate\_validate\_context\_size()
  : stan::gm
- generate\_validate\_positive()
  : stan::gm
- generate\_validate\_transformed\_params()
  : stan::gm
- generate\_validate\_var\_decl()
  : stan::gm
- generate\_validate\_var\_decls()
  : stan::gm
- generate\_var\_resizing()
  : stan::gm
- generate\_version\_comment()
  : stan::gm
- generate\_void\_statement()
  : stan::gm
- generate\_write\_array\_method()
  : stan::gm
- generate\_write\_csv\_header\_method()
  : stan::gm
- generate\_write\_csv\_method()
  : stan::gm
- get()
  : stan
- get\_base1()
  : stan::math
- get\_base1\_lhs()
  : stan::math
- get\_double\_from\_string()
  : stan::common
- get\_lp()
  : stan::math
- grad()
  : stan::agrad
- grad2F1()
  : stan::math
- grad\_hess\_log\_prob()
  : stan::model
- grad\_tr\_mat\_times\_hessian()
  : stan::agrad
  , stan::model
- gradF32()
  : stan::math
- gradient()
  : stan::agrad
  , stan::model
- gradient\_dot\_vector()
  : stan::agrad
  , stan::model
- gradIncBeta()
  : stan::math
- gradRegIncBeta()
  : stan::math
- gradRegIncGamma()
  : stan::math
- gumbel\_ccdf\_log()
  : stan::prob
- gumbel\_cdf()
  : stan::prob
- gumbel\_cdf\_log()
  : stan::prob
- gumbel\_log()
  : stan::prob
- gumbel\_rng()
  : stan::prob


---

[ Stan Home Page ]

*© 2011–2014,
Stan Development Team.*
